# Influence of slope incline on the ejection of two-phase soil splashed material

**DOI:** 10.1371/journal.pone.0262203

**Published:** 2022-01-07

**Authors:** Michał Beczek, Magdalena Ryżak, Rafał Mazur, Agata Sochan, Cezary Polakowski, Andrzej Bieganowski

**Affiliations:** Institute of Agrophysics, Polish Academy of Sciences, Lublin, Poland; Jinan University, CHINA

## Abstract

Soil splash is the first step in the process of water erosion, where impacting raindrops cause the detachment and transport of soil material. One of the factors that strongly influences the magnitude of soil splash is the incline of the surface (slope). The aim of this study was to investigate the effect of the slope on the course of the splash phenomenon caused by single-drop impact (one drop impact per soil sample), with respect to the mass and proportions of the ejected material, taking into account its division into solid and liquid phases i.e. soil and water. The investigation was carried out using three types of soil with different textures, in moistened (pressure head corresponding to -1.0 kPa) and air-dry (-1500 kPa) conditions. The soil samples were on three angles of slope, being 5°, 15°, and 30°, respectively. After a single-drop impact with a diameter of 4.2 mm, the ejected material was collected using a splash cup. The following quantities of splashed material were measured: the total mass, the mass of the solid phase, and the mass of the liquid phase. Additionally, the distribution and proportions (soil/water) of the splashed material were analysed in both the upslope and downslope directions. It was found that: (i) the change of slope had a variable influence on the measured quantities for different soils; (ii) in the case of moistened samples, the measured values were mainly influenced by the texture, while in the dry samples, by the angle of the slope; (iii) with the increase of slope, the splashed material was mostly ejected in the downslope direction (irrespective of moisture conditions); (iv) in the moistened samples, the ejected material consisted mostly of water, while in the dry samples it was soil—this occurred for material ejected both upslope and downslope. The obtained results are important for improving the physical description of the process of splash erosion. A more thorough understanding and better recognition of the mechanisms governing this phenomenon at all stages could contribute to the development of more effective methods for protecting soil against erosion.

## 1. Introduction

Soil, as the top layer of the earth’s crust and, therefore, a very important element of many ecosystems, is constantly being degraded. The causes of this degradation include various factors, which can be considered from three aspects, being chemical [1,2], biological [3,4] and physical [5,6]. Considering physical soil degradation, water erosion can be distinguished in particular [7,8]. This negative phenomenon covers a wide spectrum of processes such as the splash, surface runoff, rill and gully erosion or transport of soil material to rivers and water bodies [9,10]. Although water erosion has been the subject of research for many years, the vast majority of studies related to this phenomenon are based mainly on large-scale measurements (field experiments or catchments) [11,12] and laboratory analyses with the use of rainfall simulators [13,14]. Relatively few studies have been conducted on a micro scale, e.g., soil splash caused by the impact of a single raindrop, which is the first stage of water erosion [15,16]. During this process, caused by the impact of a single drop of water on the soil surface, there are inter alia loosening and ejection of particles (water droplets, water mixed with soil, and soil particles themselves), which are transported over different distances and in various directions [17]. Besides the ejection of particles leading to their consequent loss, the splash causes changes to the surface microstructure (a crusted surface with deterioration of infiltration parameters) [18,19] or breaking up of soil aggregates [20,21]. This phenomenon also contributes to the transfer of bacteria, fungi, and contaminants accumulated on the soil surface and subsequently displaced with the ejected particles [22,23].

There are many factors that influence the soil splash phenomenon. Generally, three groups of factors can be distinguished: a) soil properties, e.g., moisture content [24], particle size distribution [25], organic matter content [26], and characteristics of soil aggregates [27]; b) parameters of raindrops or rainfall–erosivity [28,29]; c) external factors: e.g., surface characteristics [30,31], vegetation cover [32], or soil additives [33]. Another important factor is the incline of the surface (slope) [34–36]. According to Poesen [37], raindrops cause four major effects on a hillslope: disaggregation of soil units, soil creep and lateral displacement of soil particles, saltation of soil particles into the air, and sorting of them. Soil splash studies on slopes or inclined soil samples have so far been focused mainly on measuring the mass of ejected soil transferred under the influence of rainfall, using splash cups or containers [38–40]. Considering the different angles of the slope, the magnitude of splash should be expected to vary depending on the slope direction—a bigger splash (higher mass of transferred material) will be observed downslope, and this has already been repeatedly verified in large-scale or laboratory research using precipitation simulators [35,41–43]. De Ploey and Savat [44] reported the curvilinear relationship between slope angle and particles’ detachment, which was influenced by particle size. They also observed that at low angles, grains are splashed upslope and downslope at an equal rate, resulting in a net downslope transport (upslope splash loss subtracted from the downslope splash loss) of particles close to zero. A significant correlation between the degree of the slope and a simultaneous increase in both the detachment of particles and their transport in the downslope direction (expressed by the mass of splashed soil) during rainfall experiments was presented by Quansah [45]. The effect of slope on mass and number of ejected sand grains affected by subsequent drop impacts (up to 15 impacts on sample) was investigated by Ghadiri and Payne [46]. The influence of the drop fall angle during the simulated rainfall on the amount of ejected sand particles for inclined samples was examined by Erpul et al. [47]. With the use of the cup, Nanko et al. [48] described a splash on forest litter, the substrate of which was sloping. Mati [49] investigated the splash transport of soil on a slope under various crop covers, and Ghahramani et al. [50] examined the effect of the degree of ground cover on the splash caused by rainfall on a forested slope. In a paper by Marzen et al. [51], the effect of rain and wind on the amount and distance of displaced soil on small inclination values of soil samples (0–7°) was determined using rainfall simulators and a set of gutters. Sadeghi et al. [52] noticed that slope altered the splashed particle-size distribution and the role of slope for total splashed material varied for various rainfall intensities. The influence of surface microreliefs on collected material during the splash on the slope, using splash boards, was studied by Wu et al. [53].

However, since most of the work cited above was based on studies with rainfall (natural or simulated), it is difficult to find a quantitative description of this phenomenon in the literature in relation to single-drop studies (only one drop impact per soil sample). The investigations of the single water drop impact in the context of splash provide an opportunity to investigate the initial stages and basic processes of splash erosion, which are not possible to monitor during experiments with rainfall (including simulators). Also, only such a simplified system facilitates measurements and calculation of some quantities, such as the kinetic energy of splashed particles as part of energy dissipation of the falling drop [54]. Considering the splash phenomenon on the slope, in addition to the determination of the mass of the splashed material, single drop measurements would also refer to the characteristics of the ejected particles, i.e. the proportions of the solid phase—soil and liquid phase—water. Since different types of particles are ejected during splash [17,55], it is expected that a change in the angle of the slope surface may affect the proportions and distributions of the splashed material for the downslope and upslope directions due to the various mechanisms of particle ejection. The method used in this study, based on the use of especially designed splash cups facilitating the measurement of the ratio between both phases (solid and liquid), was described in our previous work [55].

An accurate quantitative description of the splash phenomenon on a slope is very important, as inclined soil surfaces are natural conditions where the most intensive surface water erosion occurs. Considering the increasing problem of soil protection against erosion, a thorough understanding and recognition of the mechanisms governing this phenomenon at all stages could contribute to the improvement of effective methods for preventing this undesirable phenomenon. It could also enhance the development of mathematical models of splash erosion based on the physical description. Thus, the aim of the study was to investigate the influence of slope angle on the course of the splash process caused by single drop impact, especially with respect to mass and the proportions of the ejected material, taking into account its division into solid and liquid phases (soil and water).

## 2. Material and methods

### 2.1 Soil samples

The measurements were conducted on three soils belonging to different texture classes i.e. *Haplic Luvisol*, *Haplic Luvisol (Loamic)* and *Protic Regosol*. Soils were taken from the topsoil (5–20 cm) in the following mesoregions of Poland, respectively: Wysoczyzna Polanowska (54°20’06”N, 17°02’39”E), Pojezierze Dobrzyńskie (53°01’00”N, 19°12’18”E), and Wzgórza Niemczańsko-Strzelińskie (50°41’38”N, 17°00’54”E). The authors declare that no specific permissions were required for these sampling locations and confirm that the field studies did not involve endangered or protected species. The upper part of topsoil (0–5 cm) was not taken in order to remove all plant roots. The basic soil characteristics are presented in Table 1.

**Table 1:** Characteristics of soil materials.

Soil		Particle size distribution (% vol., diameter mm)						
Group (according to WRB)	Texture class	Sand 2–0.05	Silt 0.05–0.002	Clay <0.002	Initial pressure head [kPa] *		Initial water content (v %)	C_org_ [%]
*Haplic Luvisol*	loamy sand	81.6	16.6	1.8	1.0		18.97	0.91
*Haplic Luvisol (Loamic)*	sandy loam	66.2	29.7	4.1	1.0		20.39	0.67
*Protic Regosol*	silt loam	21.8	72.1	6.1	1.0		28.48	0.72

*—the pressure head values are expressed as an absolute values.

Air-dry soil samples were sieved through a 2 mm sieve and placed in aluminium rings (40 mm diameter and 10 mm height), which were secured from the bottom by a chiffon in order to prevent soil loss. The samples were prepared in two variants:

- dry (air-dry with moisture content corresponding to the pressure head -1500 kPa)–the samples were loosely packed in rings and manually levelled to obtain a smooth surface and remove soil excess,
- wet–the samples were moistened by mixing the soil with a previously measured volume of distilled water to obtain an initial moisture content corresponding to the pressure head -1.0 kPa (based on the results of a previous study [55], this pressure head was selected in order to obtain sufficiently moistened samples providing the measurable splash, i.e. the ejection of both solid and liquid phases); the surface of samples was gently smoothed (with a smooth ruler) to obtain as uniform a surface as possible.

The procedure of filling the rings was the same for all replications of all investigated soils to minimize the error caused by different water contents and bulk density of soils.

The basic information and the measured water content of soil samples are summarized in Table 1. Particle size distributions were determined using a laser diffraction method with a Mastersizer 2000 device with a Hydro G dispersion unit (Malvern, UK). The settings of the device were as follows: pump– 1750 rpm, stirrer– 700 rpm, absorption index of soil– 1.52, refractive index of soil– 0.1, aggregate disaggregation– 4 min of 35 W ultrasounds according to Bieganowski et al. [56]. Total organic carbon was determined with the Shimadzu TOC VCPH device coupled with an SSM5000 solid sample unit (Shimadzu, Japan). To make it easier for the reader to understand, the soil’s names in further parts of the study have been abbreviated as follows: HL—*Haplic Luvisol*, HLL—*Haplic Luvisol (Loamic)* and PR—*Protic Regosol*.

### 2.2 Drop generation system

The experiment was conducted with a single drop impact. Drops of water with a diameter of 4.2 mm (SD = 0.02 mm) were created using a system consisting of a peristaltic pump dosing distilled water at 20°C ± 1°C through a glass capillary. The details related to this system are described in Beczek et al. [57]. The drops fell freely from a height of 1.5 m which corresponds to an impact velocity equal to 5 m·s^-1^ and is about 60% of terminal velocity for this size of drop [58]. The kinetic energy of the drop was equal to 0.49 mJ.

### 2.3 Variants of incline

Prepared soil samples in aluminium rings were placed on a table mounted on an articulated handle to allow for different angle settings as a simulated slope. Three settings of table incline were used: 5°, 15°, and 30°. The selection of these settings results from reference to the analysis of terrain relief for the purposes of protection and landscaping of agricultural areas [59]. Taking into account the usual areas cultivated for agriculture, they are generally slopes with a slight or medium incline (up to 15°). However, other naturally occurring slopes with higher inclines, or steep slopes, where water erosion is most noticeable, should also be taken into account in the measurements.

### 2.4 Mass measurements

**Fig 1:**
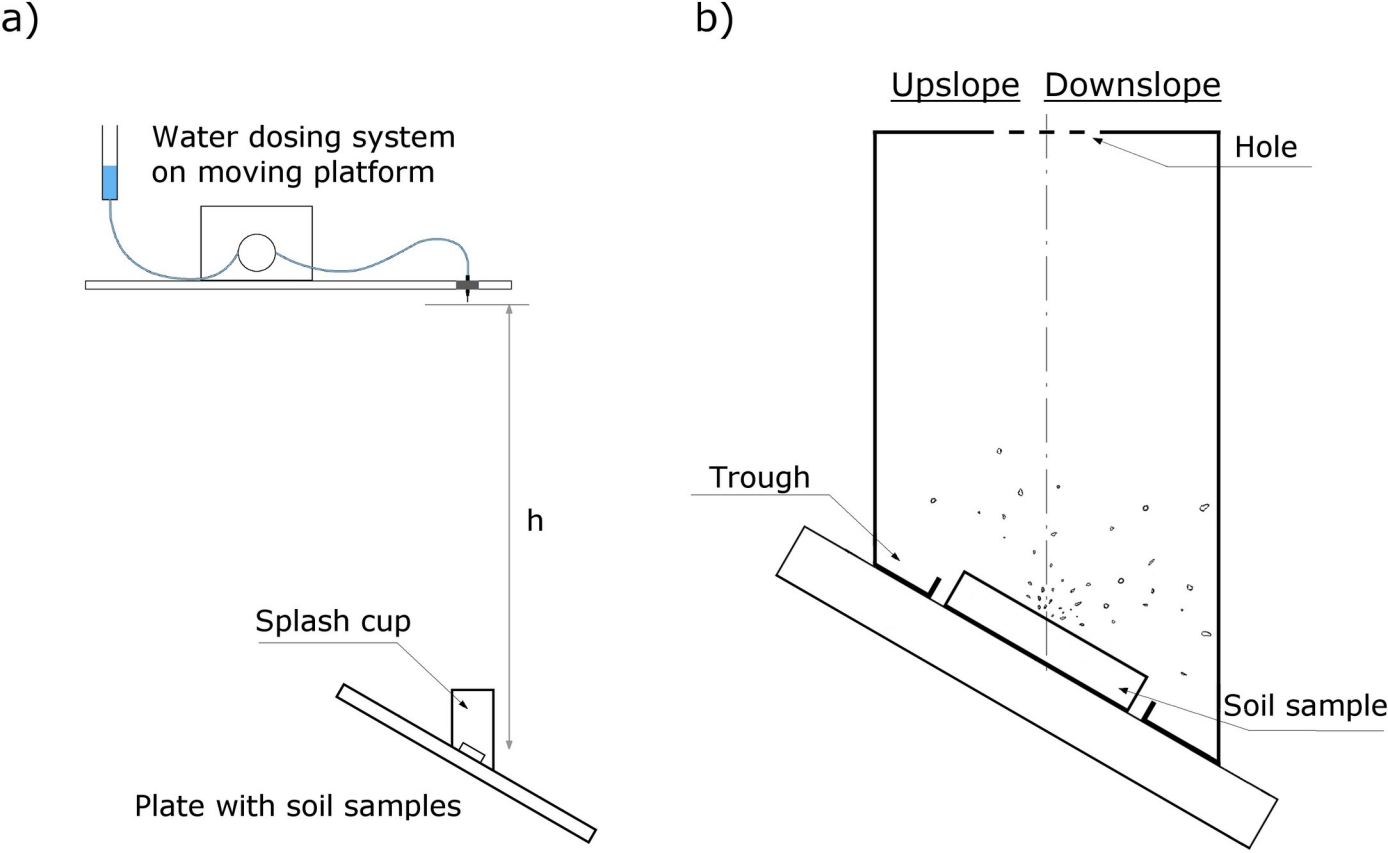
Scheme of the experimental setup (a) and drawing of the used splash cup (b). The dotted line in figure (b) marks the place where the cup is divided into the upslope and downslope parts.

Three types of particles can be ejected during the occurrence of soil splash: i) solid (i.e., soil particles or aggregates), ii) water droplets, and iii) solid particles within the water sheath (i.e., the mixture of soil and water) [17]. After a single drop impact onto the soil surface, the total ejected material was collected in a splash cup with an appropriate construction (Fig 1A), according to methodology presented by Beczek et al. [55]. The cup was made from a thin sheet of plastic film and had a round shape with a 7 cm diameter and 10 cm height (longer edge) (Fig 1B). The upper part of the cup was equipped with a hole with a 3 cm diameter for free drop impact. The bottom of the cup was inclined according to the slope setting (it was, therefore, necessary to prepare separate cups for different settings). The bottom consisted of a trough (0.7 cm height) in order to prevent the loss of collected material (especially water dripping on the walls) and a round hole (5 cm diameter) to properly set up the rings with the soil samples. The cup was divided into two parts in order to measure the ejected material for both upslope and downslope directions; however, during the drop impacts, these parts of the container were connected to each other to collect all the ejected material (including sideway ejection) and separated after the splash event to measure the upslope and downslope masses of the collected material.

Immediately after the splash, the material collected on the separate parts of the cup was weighed using an OHAUS Explorer Precision EX623 (USA) laboratory scale with 1 mg readability. This resulted in the total mass (T) of the splashed solid and liquid phases in both directions. Next, both parts of the splash cup were air dried in order to achieve a stable mass and weighed one more time to determine the mass of the solid phase (soil—M_s_). Based on this, the differences between these measured values allowed for the calculation of the mass of the splashed liquid phase (water–M_w_).

Due to the fact that the mass of splashed material after the single drop impact was small and practically immeasurable, we treated the collection of the mass from seven splash events (seven subsequent rings sample with soil) for moistened samples and three splash events for dry samples as one measurement (replication). The results were next recalculated for a single drop impact i.e. the mass collected after seven or three splash events in the splash cup (for moistened and dry samples, respectively) was divided by the number of splash events to obtain the average mass for a single drop impact per one sample. The number of splash events treated as one replication was determined by preliminary experiments. This allowed the estimation of the stable mass of collected material, which enabled the measurements to be made as quickly as possible (reducing evaporation from the collected material) and minimized the error of the mass measured in small quantities. The lower number of splash events for the dry samples was due to the greater mass collected in the splash cup from a single drop impact on the sample. It was also important to ensure the appropriate adjustment of the splash cup in relation to the ring containing the soil (centre position), thus, only the central drop impacts on the soil sample surface were taken into account in order to ensure splash symmetry. All measurements were conducted in five replications.

### 2.5 Statistical analysis

All results were subjected to analysis in STATISTICA 12 software in order to determine the statistical significance of the differences between the different soil samples and slope angles. The calculations were based on multifactorial analysis of variance (ANOVA) after checking the normality of the distributions (Shapiro-Wilk test). The statistically significant differences were determined by the post hoc test (Tukey’s HSD test) at a significance level α = 0.05. In addition, ω^2^ value calculations were performed (on the basis of the results of multifactorial analysis of variance) which, based on the Cohen scale, indicated which factors most strongly influenced the individual results.

## 3. Results

### 3.1 Masses of the ejected material

#### 3.1.1 Moistened soil samples

The quantities related to the masses of the ejected material measured with the splash cup are presented in Table 2. Analysing the moistened soil samples, the total mass (T) ranged from about 0.76 mg to 17.6 mg. The largest total mass of ejected material was achieved for the soil with the highest sand fraction content—HL. In the case of soils with higher content of fine fractions, intermediate results were obtained for HLL and the lowest for PR. The mass obtained for the HL soil was significantly higher compared to the other soils (statistically significant differences, S1 Fig). For the HLL and PR soils, increasing the degree of slope resulted in a slight increase in the total mass (especially at 30°), but statistical analysis showed no significant differences between the variants of incline. For the HL soil, the opposite trend was visible—an increase in slope gradient resulted in a significant decrease in the total mass of ejected material (S1 Fig).

**Table 2:** Masses of the ejected material obtained with a splash cup on soil samples with different inclines.

Variant	Quantity	Masses ejected after the single drop impact [mg]											
		HL			HLL				PR				
		5°	15°	30°		5°	15°	30°		5°	15°	30°
**moistened**	**T**	17.57 (C)	15.62 (C)	10.97 (D)		3.07 (AB)	3.51 (AB)	6.27 (B)		0.76 (A)	1.00 (A)	3.78 (AB)
	**M**_**s**_	7.33 (B)	7.45 (B)	4.46 (C)		0.93 (A)	0.91 (A)	1.20 (A)		0.24 (A)	0.43 (A)	0.56 (A)
	**M**_**w**_	10.24 (E)	8.17 (DE)	6.51 (CD)		2.14 (AB)	2.60 (AB)	5.07 (BC)		0.52 (A)	0.57 (A)	3.22 (BC)
**dry**	**T**	27.93 (AB)	26.47 (AB)	48.33 (C)		20.27 (A)	20.47 (AB)	38.00 (BC)		24.67 (A)	25.87 (A)	47.00 (C)
	**M**_**s**_	21.20 (AB)	19.33 (AB)	35.27 (C)		15.80 (AB)	11.53 (A)	25.27 (BC)		18.93 (AB)	19.07 (AB)	35.27 (C)
	**M**_**w**_	6.73 (A)	7.13 (AC)	13.07 (B)		4.47 (A)	8.93 (ABC)	12.73 (B)		5.73 (A)	6.80 (A)	11.73 (BC)

The values are recalculated for a single drop impact. Symbols of measured quantities: T–total mass of ejected material; M_w_−mass of ejected liquid phase (water); M_s_−mass of ejected solid phase (soil). Symbols of soils: HL–*Haplic Luvisol*, HLL–*Haplic Luvisol Loamic*, PR–*Protic Regosol*. The letters in parentheses refer to the statistical comparison between investigated soils and inclines for the measured quantities (the same letters in row–no statistically significant differences).

In the case of the ejected solid phase (soil)—M_s_, similar trends as above were noted for all investigated soils (Table 2). As before, the highest mass of splashed soil was noted for HL, regardless of slope. A slight increase in mass was observed for HLL and PR soils (but no statistically significant differences were noted), and a significant decrease for HL soil with the slope equal to 30° (S2 Fig).

Based on the results for the ejected liquid phase—M_w_, in each case, the mass of splashed water was greater than the mass of splashed soil (Table 2). The trends were similar to the ejected solid phase, but the increase in the mass of water was much more apparent for HLL and PR soils when the slope changed to 30°. An interesting observation may be that despite the decreasing mass for HL and increasing masses for HLL and PR with increasing slope, at a slope of 30° the mass of splashed water in all cases was relatively similar, and no statistically significant differences were noted (S3 Fig).

#### 3.1.2 Dry soil samples

Considering the dry samples (Table 2), the total mass of splashed material (T) was notably greater than for the moistened samples, ranging from about 20.3 mg to 48.3 mg. Regardless of the analysed soils, the ejection of particles was similar in all cases, hence, no statistically significant differences between soils at different surface inclines were observed. For 5° and 15° slopes, the measured masses ranged from approximately 20 mg to 28 mg, but for all soils, there were no statistically significant differences (S6 Fig). The increase of the slope to 30° resulted in a substantial increase (almost double) in total ejected mass for all investigated soils.

In the case of the splashed solid phase (M_s_), similar trends were noted for all soils regarding the total mass (Table 2). The mass of soil that was ejected ranged from approximately 13 mg to 35 mg. As before, for slopes of 5° and 15°, the masses of the solid phase were similar for all soils (no statistically significant differences–S7 Fig). Changing the slope to 30° resulted in an increase in mass for all soils.

In each case, the mass of the splashed water (M_w_) was several times lower than the mass of the ejected soil (Table 2). A slight increase in the mass of water was observed for all soils with an increase in slope from 5° to 15°, but no statistically significant differences were noted (S8 Fig). A definite increase was observed at a slope of 30°, but for all soils, the mass was very similar. For all of the observed results in the case of dry samples, the mass of transferred water consisted of between 11% to 34% of the mass of the falling drop.

### 3.2 Proportions of the ejected material

#### 3.2.1 Moistened soil samples

**Fig 2:**
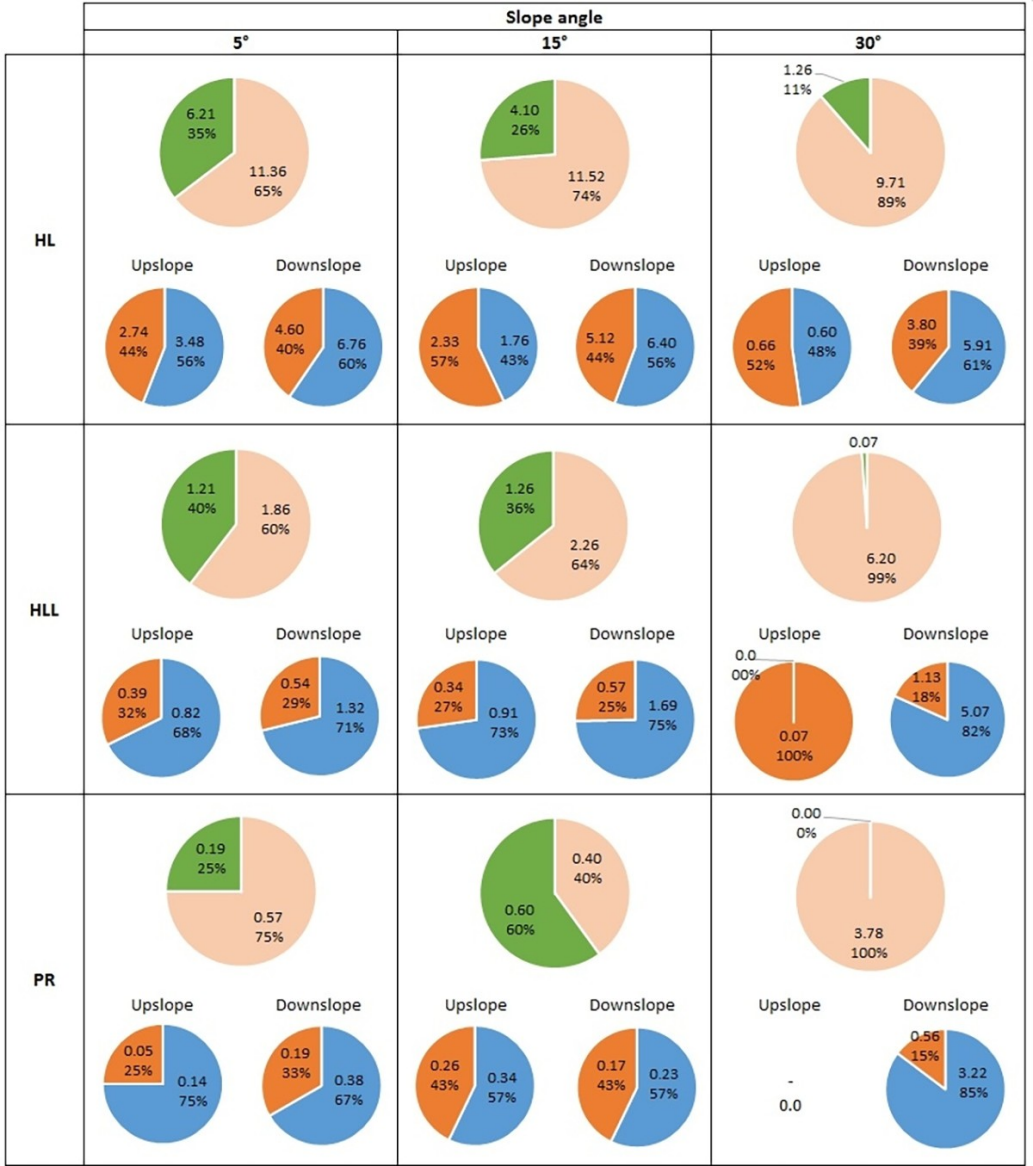
Proportions of the ejected material on moistened soil samples with different inclines. The upper graph shows the total material ejected into the upslope (green colour) and downslope (slight beige colour) directions. The lower graphs present the proportions of soil (brown) and water (blue) in the ejected material for both directions. Symbols of soils: HL–*Haplic Luvisol*, HLL–*Haplic Luvisol Loamic*, PR–*Protic Regosol*. All values are expressed in [mg] units.

**Fig 3:**
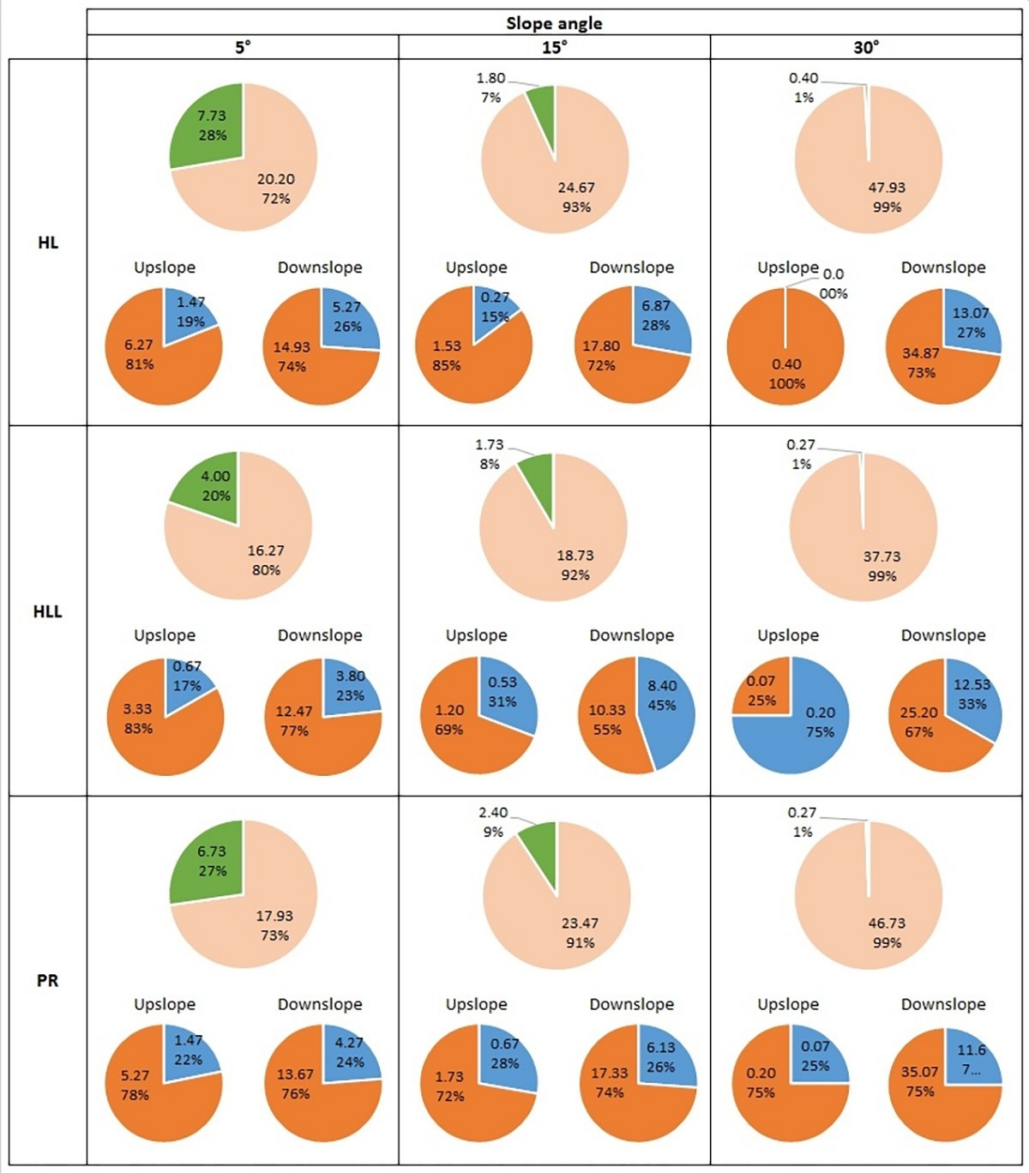
Proportions of ejected material on dry soil samples with different inclines. The upper graph shows the total ejected material in the upslope (green colour) and downslope (slight beige colour) directions. The lower graphs present the proportions of soil (brown) and water (blue) in the ejected material for both directions. Symbols for soils: HL–*Haplic Luvisol*, HLL–*Haplic Luvisol Loamic*, PR–*Protic Regosol*. All values are expressed in [mg] units.

The total mass distribution and proportions (with division into soil and water) of the splashed material in both upslope and downslope directions is presented in Figs 2 and 3.

Analysing the mass distribution for moistened samples (upper graphs on Fig 2), generally, in the case of all investigated soils for 5° and 15° slopes, the distribution looked similar, i.e., about 25–40% of the ejected material was transferred on the upslope and about 60–75% on the downslope. The exception was the PR variant at 15°, where more material was ejected in the upper direction, but there were no statistically significant differences between both directions (S4 Fig). Changing the slope to 30° resulted in about 99% of the splashed material being directed downslope and only 1% in the upslope direction for the HLL soil, while 100% of the material was moved in the downslope direction for the PR soil. For the HL soil on a slope of 30°, as much as 89% of the material was displaced in the downslope direction, while in contrast to the other soils, 11% of the material was displaced in the upward direction. Referring to the absolute mass caught in the upslope direction (S4A Fig), the greatest decreases (the biggest changes) for successive variations in slope were noted for HL soil.

Considering the proportions of the ejected material when divided into splashed water and soil (lower graphs on Fig 2), in almost every case (regardless of the upslope/downslope direction), the proportion of material was in favour of water which accounted for 56–85%. The exceptions were the HL soil at 15° and 30°, where, in the upslope direction, the material consisted slightly more of ejected soil (but there were no statistically significant differences between solid and liquid phases; S5 Fig). For the HLL and PR soils, the 30° slope was notable in that 100% of the ejected soil was recorded in the upslope direction for HLL (but this was a small mass of only 0.07 mg), and for the PR soil, it was impossible to determine the proportions due to the lack of recorded mass (probably very low mass of ejected droplets).

#### 3.2.2 Dry soil samples

Looking at the results obtained for dry samples, for all analysed soils, the mass of ejected material was mainly displaced in the downslope direction and accounted for approximately 72–80% at 5° incline, increasing to 99% at 30° incline (upper graphs on Fig 3). In all soils, for the upslope direction, the percentage contribution in total ejected mass slightly decreased as the slope increased. Unsurprisingly, the opposite trend was seen for the downslope direction–in the case of the 5° and 15° slopes, the increase was small, and no statistically significant differences were observed, but changing the slope to 30° immediately resulted in a considerable increase in mass.

Analysing the proportions of ejected material divided into splashed water and soil (lower graphs on Fig 3), as previously mentioned, for dry samples, the ejected material consisted primarily of soil. Regardless of soil texture and slope, this accounted for 55% to 85% (or even 100% for HL soil at 30° slope in the upslope direction). The exception was the HLL soil at 30°, where, for the upslope direction more ejected water was registered, however, there were no statistically significant differences between either phase (S10 Fig). Referring to the absolute masses presented in S10 Fig, in the case of ejected soil in the upslope direction, for all soils samples the registered mass was similar and decreasing with more inclined slopes (there were no statistically significant differences between soils). In contrast, for the downslope direction, the mass of splashed soil was significantly larger and increased with increasing slope. In the case of ejected water, the trends looked similar. In the upslope direction for all soils, the mass of splashed water was similar and decreased with increase in slope (no statistically significant differences). However, it should be noted at this point that these results were very small values–from 0 to 1.47 mg. For the downslope direction, there was an increase in the mass of water, but when comparing both directions, statistically significant differences were visible for the 15° and 30° slopes.

### 3.3 Statistical influence of factors

The results presented in subchapters 3.1 and 3.2 were subjected to statistical analysis of ω^2^ value which, based on the Cohen scale, indicated to what extent either individual factors or groups of factors influenced the results of the measured quantities. The results of this analysis are presented in Table 3.

**Table 3:** Values of ω^2^ indicating to what extent individual factors or groups of factors influenced the results of the investigated quantities.

**Moistened soil samples**					
**Factors**	**Total ejected mass (T)**	**Mass of soil (M**_**s**_ **)**	**Mass of water (M**_**w**_ **)**	**Mass proportions in upslope and downslope**	**Proportions of ejected material (soil/water)**
soil texture	0.819	0.827	0.726	0.493	0.425
slope (inclination)	-0.003	0.009	0.011	-0.002	-0.001
direction (upslope/downslope)				0.196	0.169
material (ejected soil/water)					0.037
soil * slope	0.087	0.044	0.124	0.052	0.046
soil * direction				0.091	0.079
slope * direction				0.041	0.036
soil * material					0.002
slope * material					0.007
direction * material					0.026
soil * slope * direction				0.004	0.005
soil * slope * material					0.003
soil * direction * material					0.000
slope * direction * material					0.011
soil * slope * direction * material					0.002
**Dry soil samples**					
**Factors**	**Total ejected mass (T)**	**Mass of soil (M**_**s**_ **)**	**Mass of water (M**_**w**_ **)**	**Mass proportions in upslope and downslope**	**Proportions of ejected material (soil/water)**
soil texture	0.078	0.131	-0.007	0.011	0.007
slope (inclination)	0.674	0.545	0.614	0.090	0.062
direction (upslope/downslope)				0.651	0.451
material (ejected soil/water)					0.133
soil * slope	-0.016	-0.013	0.007	-0.002	-0.001
soil * direction				0.004	0.003
slope * direction				0.183	0.127
soil * material					0.008
slope * material					0.012
direction * material					0.078
soil * slope * direction				0.001	0.001
soil * slope * material					0.000
soil * direction * material					0.004
slope * direction * material					0.025
soil * slope * direction * material					0.001

The values were calculated based on multifactorial analysis of variance. Colours of labels indicate the strength of the effect: red–no effect, yellow–low effect, blue–medium effect, green–high effect, gray–N/A (not applicable).

Considering the moistened soil samples, all of the investigated quantities were influenced mainly by the soil texture factor (high or medium effect). In the case of the mass of ejected water (M_w_), a low impact of the combination of soil texture and slope was noticeable. For both quantities related to proportions of ejected material, a minimal effect resulting from the direction of ejection was observed.

On the other hand, in the case of the dry soil samples, a high effect of slope was noted for total ejected mass (T), mass of ejected soil (M_s_), and mass of water (M_w_). A high or medium influence of the direction of ejection was visible for quantities related to proportions. Also, for these measured values, a low effect of the combination of slope and direction was observed.

## 4. Discussion

### 4.1. Energy of a water drop hitting the soil surface

As mentioned in the ‘Materials and methods’ section, the height from which the drop fell was 1.5 m and the resultant impact velocity was about 60% of the terminal velocity of a water drop of this size. However, it should be noted that we intentionally used a bigger water drop. During medium and intensive rainfalls the mean size of the falling drops is usually slightly over 2 mm [60,61]. The terminal kinetic energy of such raindrops is about 0.1 mJ. Our bigger water drop (4.2 mm in diameter), which was falling from “only” 1.5 m, had the energy equal to 0.49 mJ, i.e. almost 5 times greater. This energy was equivalent to the water drop of a ca. 3-mm diameter, which achieves the terminal velocity. What is more, water drops of 4.2 mm used in this study are observed in natural rain events [62,63].

Only the central hitting of the water drop in the rings with the soil sample were interpretable and only such cases were included in the processing of the results, which is important in single-drop experiments. However, the greater height of the falling drop is a more advanced technical issue requiring precision in the place of hitting. The use of the bigger size of the water drop and the smaller height of the fall enabled us to observe the splash phenomenon, which is comparable with the natural conditions, avoiding technical problems.

However, there is one more argument in favour of using the height of 1.5 m with a drop size of about 4 mm. Heights and water drop sizes of such a magnitude are observed in the throughfall phenomenon when the drops fall from trees and plants [64,65]. This phenomenon is especially important in forests, as the soil surface under trees is often not covered by any plants and is therefore more susceptible to erosion than soils under the direct impact of raindrops but covered by plants.

### 4.2. Masses of the ejected material

When discussing the amount of material being ejected during the splash, as presented in Tables 2 and 3, it should be born in mind that the total mass of ejected material (T) is dependent on the mass of ejected liquid phase (water—M_w_) and the mass of ejected solid phase (soil—M_s_). However, because the ratio of both phases of ejected material can differ, it should be expected that this dependence would not be the same in all investigated variants. To begin the discussion, it is worth citing the results presented in work by Beczek et al. [55], wherein measurements were made on horizontal surfaces, without a slope (i.e., at an angle of 0°), using samples prepared from the same soils as in this study. The general observations were very similar: the largest amount of ejected material was noted for loamy sand soil (HL) and the least for silt loam soil (PR). The total ejected masses obtained on flat surfaces for those soils were consistent with masses achieved on the lowest incline of slope (5°) for moistened samples in this work.

In analysing the mass of ejected material (total and also for solid and liquid phases) for moistened soils relative to the slope, the most important observation is that the trend for HL soil was opposite to the other two soils. Mizugaki et al. [66] stated that the angle of slope induces different soil compaction as a result of the impact of a drop, which seems to be one of the issues that could be considered here. According to the authors, the highest pressure is exerted when the falling drop hits the soil surface perpendicularly and, the greater the angle of the slope, the lower the pressure created by the impacting drop. As a consequence–the lower pressure results in less ejected material. This observation is consistent with the work by Erpul et al. [67], who found that raindrop impact pressure decreased by 35% during rainfall events as the slope angle increased from 27° to 34°. However, if such an explanation is valid, the question then arises as to why for two other soils (i.e., HLL and PR) the opposite trend was observed—the greater the angle of the slope, the greater the amount of ejected material.

The explanation seems to be the particle size distribution, or rather, as a result, the pore size distribution and soil surface conditions. Due to the loose structure and large pores in HL soil, water can move more freely between them in the moistened sample. Beczek et al. [55], previously suggested that on a moistened HL soil, a layer of water appears on the surface due to the above-mentioned structure. This led to the formation of the so-called crown phenomenon (formed from a mixture of water and soil) contributing to the subsequent ejection of large amounts of material. In this study, the water content in the moistened samples was high– 1 kPa of pressure head means that the soil is close to saturation. Moreover, the soil surface incline caused the movement of water from inside the soil sample towards the slope surface.

This movement towards the surface can be explained as follows: under the influence of gravity, the water in the entire sample flowed down into the larger pores. If there was no slope, after some time, an equilibrium would be established, and the moisture profile would be stabilized. However, the water close to the slope could be flowing in the direction of the slope, because the flow resistance towards the slope in the surface layer would be the lowest, resulting in the amount of water on the slope surface being greater than the theoretically prepared. How different? We are not able to assess this accurately, we can only state that the bigger the slope, the bigger the surface water content, and that we did not observe any water flowing out of the soil. This confirms the development of suitable conditions for the formation of the crown phenomenon. The arguments for this reasoning being: i) when the pores in the soil are smaller and the movement of water is, therefore, not so easy; the phenomena observed in HL was not seen in either of the other soils containing more finer fractions (i.e., HLL and PR); ii) the statistical analysis showing that the texture of the soil had the main influence on the mass of ejected material in moistened samples (Table 3); iii) considering the dry samples, the trend in HL was the opposite (compared to moistened samples) and the same as in both other soils. According to the available literature, it could be expected that as the slope increased, there would be a change in the formation of the crown and the material ejected by it [46,68]. A theory that during the drop impact onto an oblique surface, tangential velocity occurs, making the crown shape asymmetrical was presented by Roisman and Tropea [69]. This was confirmed by Bird et al. [70], who found that the asymmetry leads to an azimuthal variation of the ejected rim and the tangential component of impact can suppress a splash, causing faster crown rupture and reducing the number of ejected droplets. Some experiments showed that on the slope surface, the rim of the forming crown is much higher on the downslope side than on the upslope, which leads to the ejection of more droplets on the downslope [71,72]. In our study, the lowest incline (5°) resembled conditions close to a horizontal surface, without causing significant changes in crown formation and ejection of material. But increasing the slope may have already substantially affected this phenomenon, causing a decreasing trend in the mass of total ejected material. However, it should be remembered that the works mentioned above mainly concern the drop impact phenomenon on model oblique surfaces (aluminium/titanium with a water layer) and it was not possible to find such works in relation to soil samples.

To clarify the reverse trend for HLL and PR soils one more aspect should be considered–the cohesion resulting from particle size distribution. It is well known that in sandy soil, the cohesion is negligible compared to those soils containing larger amounts of finer fractions [73]. HLL and PR soil samples, due to their greater content of fine fractions (especially PR soil) and with adequate moisture content (1.0 kPa), have higher cohesion compared to HL soil. Thus, the detachment and ejection of particles are definitely more difficult from the surface of the soil samples, and the mass of total ejected material was, therefore, lower (Table 2). This has been shown previously in work by Beczek et al. [55], during splash experiments on horizontal surfaces. However, changing the incline of the slope slightly increased the mass of ejected material. This may be due to the aforementioned higher cohesion (and plasticity) of the surface, which results in a greater contribution to the ejection of water coming from the impacting drop. With the increased slope (especially at 30°), the drop exerts less pressure on the surface (which results in limited penetration) and breaks up to a greater extent into small droplets transferred onto the splash cup. This is confirmed by the results shown in Table 2 (and also S2 and S3 Figs), where no change in the mass of ejected soil and a definite increase in the mass of ejected water with the change in slope were noticeable for HLL and PR soils, suggesting that the liquid phase may have had a decisive effect on the total ejected mass.

Analysing the dry samples, the general observation for all soils was that the bigger the angle of slope, the greater the amount of ejected material (including separate solid and liquid phases). Such an observation runs against the above-mentioned reasoning of Mizugaki et al. [66], but these authors only investigated natural hillslopes (natural moisture conditions). However, the influence of cohesive force is a good visible guide instead. The air-dried soil samples, sieved through 2 mm mesh, investigated in this study resemble loose materials in which cohesion is almost non-existent [74], allowing for easy detachment and ejection of the soil particles. Thus, in contrast to the moistened samples, here we have a uniform trend for all soils. It is noteworthy that in the case of the mass in the ejected liquid phase (Table 2 and S8 Fig), its values were considerably lower than those in the ejected solid phase (facilitated ejection of grains and soil aggregates) (S7 Fig). The transferred water derived entirely from the impacting drop and surprisingly, the values were higher compared to those of the moistened samples (but in that case, it was not possible to indicate to what extent the water was from the drop and to what extent from the soil sample). The noticeable increase in the mass of the liquid phase with increasing slope can probably be explained by the way in which the falling drop is broken up into tiny droplets and displaced on differently sloped surfaces, where at higher slopes its momentum causes the droplets to be moved more easily in the downward direction, as confirmed by S10 Fig This seems to be supported by the work of Brodowski [75], in which it was shown that for a higher incline, higher values of the (vector) components of drop velocity follow the direction of the surface slope. Thus, ejected water droplets originating from a falling drop are more easily moved downward.

Although it was possible to compare our results with the works describing the influence of the slope on the mass of transferred material in the general categories (referenced below), the comparison of masses of transferred materials caused by single drop impact was impossible, largely because we were unable to find such publications. Referring to the available literature on similar research, using different soils, five slope gradients, and various rainfall intensities, Quansah [45] found that the most important factor that influenced splash detachment and splash transport was the soil type and then rainfall intensity with the slope and the rainfall intensity, respectively. This observation is in line with our results for moistened samples where the soil texture was a more important factor than the slope. Ghadiri and Payne [46] checked the effect of the slope on the amount (mass) of splashed material in their investigations of subsequent single drop impacts (from 10 to 15) onto coarse sand. They noticed the increasing total collected mass with the increasing slope angle resulting from the increasing mass for the downslope direction (up to 117 mg). For the upslope direction, the trend was reversed–the greater the angle, the lower the mass of ejected sand, and the upper direction was no longer relevant from the angle between 30–40°. It should be noted that the authors collected significantly higher masses of ejected material (up to 122 mg of total mass) in comparison to our results. However, in their experiments, they used drops with a larger diameter (6.2 mm) falling from 4 m, which resulted in higher kinetic energy, and used from 10 to 15 drop impacts in single experiments. Liu et al. [43] investigated the effect of slope gradient on raindrop splash erosion with the use of a rain simulator and soil trays simulating nine gradients of slope. The authors observed that downslope splash erosion, total splash detachment, and net splash transport increased initially with rising slope, and then decreased at the critical slope gradient, being 35°, all showing linear relationships. Other authors performed experiments to check the influence of rainfall intensity and slope gradient on splash from a saline–sodic soil under coastal reclamation [76]. Based on their results, the total splash increased to maximum levels as the slope increased to 11° but decreased with further increases in gradient. However, the comparison of these results with ours can only be qualitative because the results of the splash analysis with the use of a rain simulator are not the simple sum of many single-drop splashes (including the changes in soil water content).

### 4.3. Proportions of the ejected material (upslope/downslope)

It is not surprising that much more of the material is ejected downslope than upslope (Figs 2 and 3), which was also observed in other works [43,35,67,77]. However, to the best of the authors’ knowledge, such deep analysis in relation to a single-drop splash (measured while taking into account the phase of the transported material) is presented here for the first time. Generally, the greater the slope, the less material was ejected upslope (Figs 2 and 3). This issue had been theoretically considered by Brodowski [75]. The author reported that with the increase of slope to about 20°, the distribution of the kinetic energy of the impacting drop which caused the ejection of the soil particles is divided in the ratio 25:75, meaning that about 25% of the energy is responsible for transporting the material upslope, and about 75% for downslope transport.

On moistened samples, regardless of the differences between the soils, the course of the splash on 5° and 15° slopes was similar. Only the 30° slope caused visible (and statistically significant) differences. The considerations of Brodowski [75] presented in the above paragraph are of course valid in this case; however, other factors also start to play a role in this phenomenon such as the angle of the particles’ ejection in relation to the angle of the slope. In order to eject soil particles and/or water droplets upslope, it is necessary for the ejection angle to be greater than the incline of the slope. In case of dry samples, for each of the investigated soils, the differences for varying slopes were more visible. An interesting observation might be that for the downslope direction, an increase from 15° to 30° resulted in a significant increase in the mass of the ejected material (more than double). For the upslope direction, this incline may be considered as a certain limit at which this direction still played a role due to the fact that, for all investigated soils, the mass ejected upslope constituted only 1% of the total mass.

When comparing our results with other works, we are more consistent with the work of Froehlich [41], who observed that more ejected material was splashed downslope than upslope. Liu et al. [43] investigated the splash erosion on a slope with the division into upslope, downslope, and lateral directions, and observed that downslope splash erosion varied more notably than the others, which indicates that the downslope direction is more significantly affected by slope gradient. Fu et al. [42] indicated that upslope splash loss was a very important component of the total splash loss on gentle slopes and may be neglected on slopes greater than 36%. It is difficult to relate this directly with our study, due to the scale of the investigated phenomenon; however, those results are consistent with our results presented in Table 2, S4 and S8 Figs It is also worth noting that for the moistened HLL and PR samples in our work, changing the slope inclination to 30° compared to other values resulted in an upslope splash that was almost completely inhibited, which was due to the aspects mentioned above as well as the cohesion properties of these soils. A similar trend was observed by Liu et al. [76], who investigated the splash effect on saline-sodic soils, noting that the maximum value of upslope splash occurred when the slope gradient was 22° and then decreased. Such an observation appears to be partially consistent with the results for moistened HLL and PR samples, for which the highest values of upslope splash was observed at 15°.

### 4.4. Proportions of the ejected material (soil/water)

It can be stated that the proportions of water mass to the total mass of ejected material was more or less constant in all cases where the ejected mass was measurable. The differences only varied by plus/minus a few per cent but there were no visible trends with changing slope. The only general conclusion can be that in the moistened samples, the ejected material consisted mostly of water while in the dry samples, it was soil—this occurred both for material ejected upslope and downslope. In the case of the moistened samples, the detachment and ejection of particles required significantly more energy than in the dry samples because of the higher cohesion. Thus, a small amount of solid phase was displaced and most of the mass of ejected material probably represented water from the impacting drop. It should be remembered that in this case, the splashed liquid phase was probably also the water from the sample (especially in low-cohesion HL soil), but it is difficult to indicate to what extent.

It is very difficult to refer to other works in terms of the proportion of ejected material separated into water and soil, as such studies are uncommon. With respect to horizontal surfaces, in the work of Beczek et al. [54], we can find splash investigation on the same soils (with similar moisture content) and the determination of the mass of solid and liquid phases in the ejected material. For soils with a high content of fine fractions (HLL and PR soils in this study), the proportions were about 76–81% of the splashed water and about 19–24% of the splashed soil, while for sandy soil (HL), the proportions were 62% (water) and 38% (soil). Comparing this to the results in our work, these values are very close to the proportion of the ejected material for moistened samples with a surface inclined at 5° (whether for the downslope or upslope direction).

## 5. Conclusions

The applied methodology, based on the use of a splash cup, allowed for the characterization of the ejected material during the splash phenomenon caused by the impact of a single drop on the slope surface. The following quantities were measured: the total mass of the ejected material, divided into the mass of the solid phase (soil) and the mass of the liquid phase (water). Additionally, the distribution and proportions of the splashed material were analysed in both the upslope and downslope directions. The change of these values for 3 different variants of surface slope was investigated in this study.

In the case of moistened soil samples, the texture of the investigated soils and the resulting cohesion, as well as the surface condition, had a significant influence on the measured quantities. The highest amounts of ejected material were obtained for soil with the highest content of sand fractions, and the lowest for soil with the highest content of fine fractions. With the increasing slope, no uniform trend was observed for all investigated soils; for the soil with the highest content of sand fraction, an increase in slope caused a decrease in total mass, while for soils with a higher content of fine fractions, mass gently increased, especially at an incline of 30°. Similar trends were noted for the mass of ejected water and ejected soil. As far as the amounts of splashed material in the upslope and downslope directions were concerned, they were very similar for each of the investigated soils, both at 5° and 15° slopes, with most of the material ejected downward (60–75%). Whereas, in the case of a slope of 30°, for soils with a higher content of fine fractions, practically all the material was ejected in the downslope direction, and for soil with the highest sand content, the upslope direction was noticeable. Analysing the proportions of splashed material (water/soil), in almost every case (regardless of whether an upslope or downslope direction) they were in favour of the ejected water.

For the dry samples, soil texture was no longer of major importance and differences in measured quantities between soils were statistically insignificant, making the observed trends similar for all. However, the effect of slope was significant; as the incline increased, the mass of ejected material increased, and a dynamic increase was seen at a slope of 30°. Such a trend was seen for both total mass of ejected material, and separated masses of ejected soil and water. Considering the distribution of splashed material in the upslope and downslope directions, similar to the moistened samples, the vast majority was displaced downslope (ranging from about 70% at 5° to 99% at 30°). Whereas, when taking into account the proportions of the material, regardless of soil texture and slope angle, it accounted for 55% to 85% in favour of ejected soil.

## Supporting information

S1 Fig Total ejected material on moistened soil samples for different slope angles. Symbols of soils: HL–*Haplic Luvisol*, HLL–*Haplic Luvisol Loamic*, PR–*Protic Regosol*. The bars represent standard error, and the letters refer to the statistical comparison (the same letters–no statistically significant differences).
(TIF)

S2 Fig Mass of ejected solid phase (soil) on moistened soil samples for different slope angles. Symbols of soils: HL–*Haplic Luvisol*, HLL–*Haplic Luvisol Loamic*, PR–*Protic Regosol*. The bars represent standard error and letters refer to the statistical comparison (the same letters–no statistically significant differences).
(TIF)

S3 Fig Mass of ejected liquid phase (water) on moistened soil samples for different slope angles. Symbols of soils: HL–*Haplic Luvisol*, HLL–*Haplic Luvisol Loamic*, PR–*Protic Regosol*. The bars represent standard error and letters refer to the statistical comparison (the same letters–no statistically significant differences).
(TIF)

S4 Fig Mass of total ejected material in upslope (a) and downslope (b) directions on moistened soil samples with different slope angles. Symbols of soils: HL–*Haplic Luvisol*, HLL–*Haplic Luvisol Loamic*, PR–*Protic Regosol*. The bars represent standard error and letters refer to the statistical comparison (the same letters–no statistically significant differences). The statistical analysis allows the comparison of both graphs.
(TIF)

S5 Fig Mass of separate ejected soil (a) and ejected water (b) in upslope and downslope directions on moistened soil samples with different slope angles. Symbols of soils: HL–*Haplic Luvisol*, HLL–*Haplic Luvisol Loamic*, PR–*Protic Regosol*. The bars represent standard error and letters refer to the statistical comparison (the same letters–no statistically significant differences). The statistical analysis allows the comparison of all visible graphs.
(TIF)

S6 Fig Total ejected material on dry soil samples for different slope angles. Symbols of soils HL–*Haplic Luvisol*, HLL–*Haplic Luvisol Loamic*, PR–*Protic Regosol*. The bars represent standard error and letters refer to the statistical comparison (the same letters–no statistically significant differences).
(TIF)

S7 Fig Mass of ejected solid phase (soil) on dry soil samples for different slope angles. Symbols of soils: HL–*Haplic Luvisol*, HLL–*Haplic Luvisol Loamic*, PR–*Protic Regosol*. The bars represent standard error and letters refer to the statistical comparison (the same letters–no statistically significant differences).
(TIF)

S8 Fig Mass of ejected liquid phase (water) on dry soil samples for different slope angles. Symbols of soils: HL–*Haplic Luvisol*, HLL–*Haplic Luvisol Loamic*, PR–*Protic Regosol*. The bars represent standard error and letters refer to the statistical comparison (the same letters–no statistically significant differences).
(TIF)

S9 Fig Mass of total ejected material in upslope (a) and downslope (b) directions on dry soil samples with different slope angles. Symbols of soils: HL–*Haplic Luvisol*, HLL–*Haplic Luvisol Loamic*, PR–*Protic Regosol*. The bars represent standard error and letters refer to the statistical comparison (the same letters–no statistically significant differences). The statistical analysis allows the comparison of both graphs.
(TIF)

S10 Fig Mass of separate ejected soil (a) and ejected water (b) in the upslope and downslope directions on dry soil samples with different slope angles. Symbols of soils: HL–*Haplic Luvisol*, HLL–*Haplic Luvisol Loamic*, PR–*Protic Regosol*. The bars represent standard error and letters refer to the statistical comparison (the same letters–no statistically significant differences). The statistical analysis allows the comparison of all visible graphs.
(TIF)

S1 Graphical abstract (TIF)
