# Supplementary figures and images for: Influence of slope incline on the ejection of two-phase soil splashed material
Source: PLoS One. 2022 Jan 7;17(1):e0262203. doi: 10.1371/journal.pone.0262203 (PMC8741056; doi:10.1371/journal.pone.0262203)

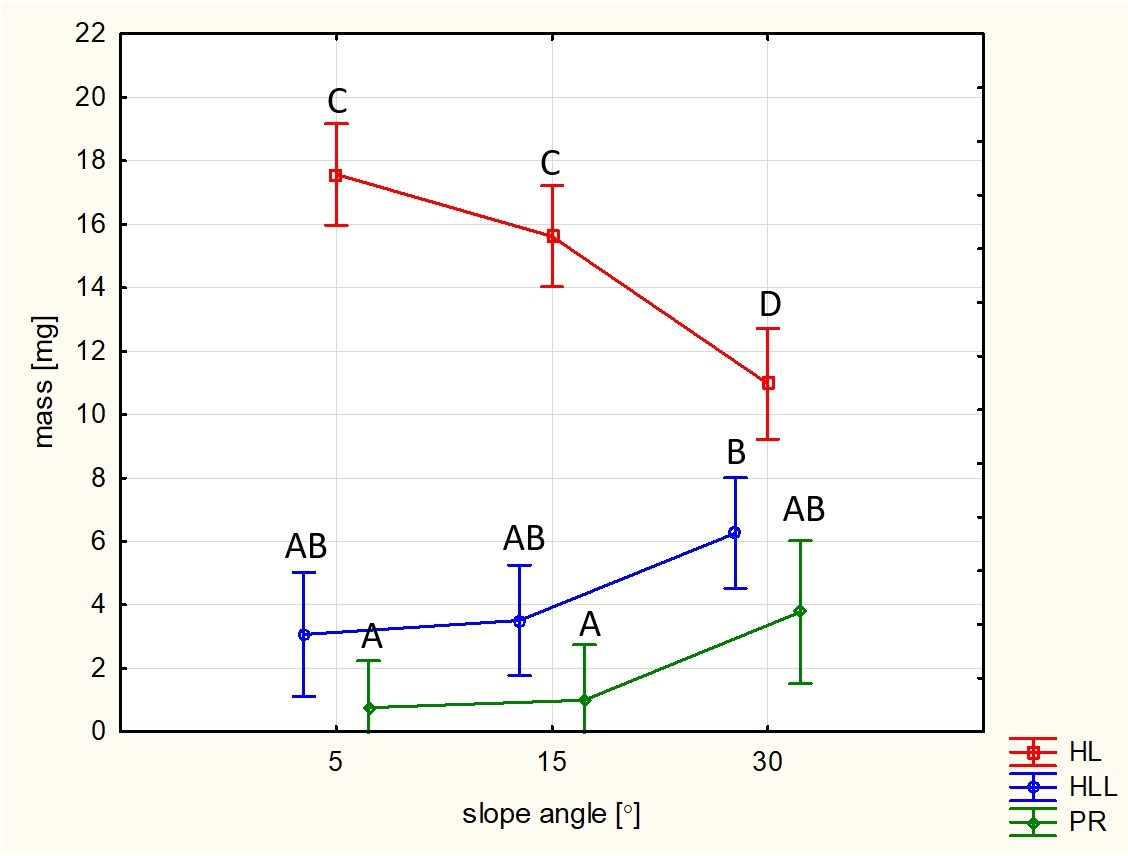

Supplement: S1 Fig — Symbols of soils: HL–Haplic Luvisol, HLL–Haplic Luvisol Loamic, PR–Protic Regosol. The bars represent standard error, and the letters refer to the statistical comparison (the same letters–no statistically significant differences). (TIF) [file pone.0262203.s001.tif]

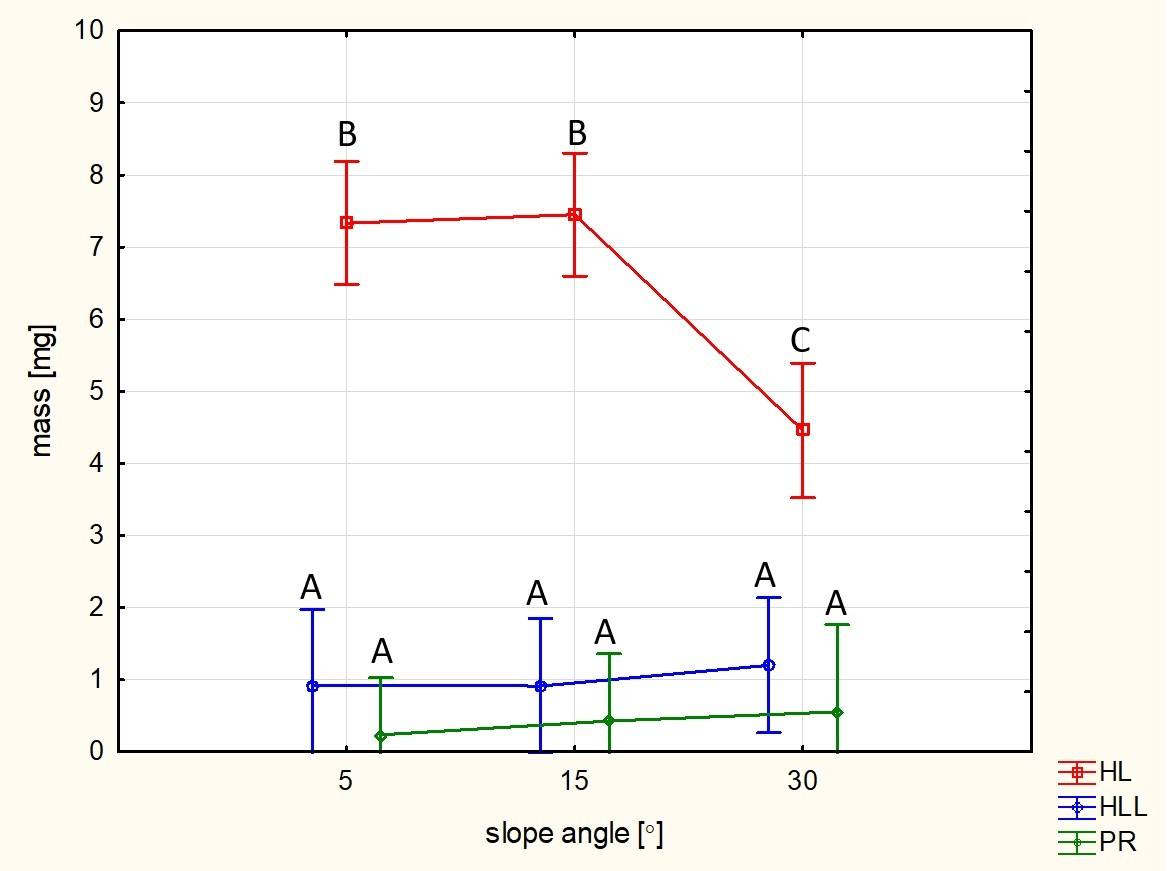

Supplement: S2 Fig — Symbols of soils: HL–Haplic Luvisol, HLL–Haplic Luvisol Loamic, PR–Protic Regosol. The bars represent standard error and letters refer to the statistical comparison (the same letters–no statistically significant differences). (TIF) [file pone.0262203.s002.tif]

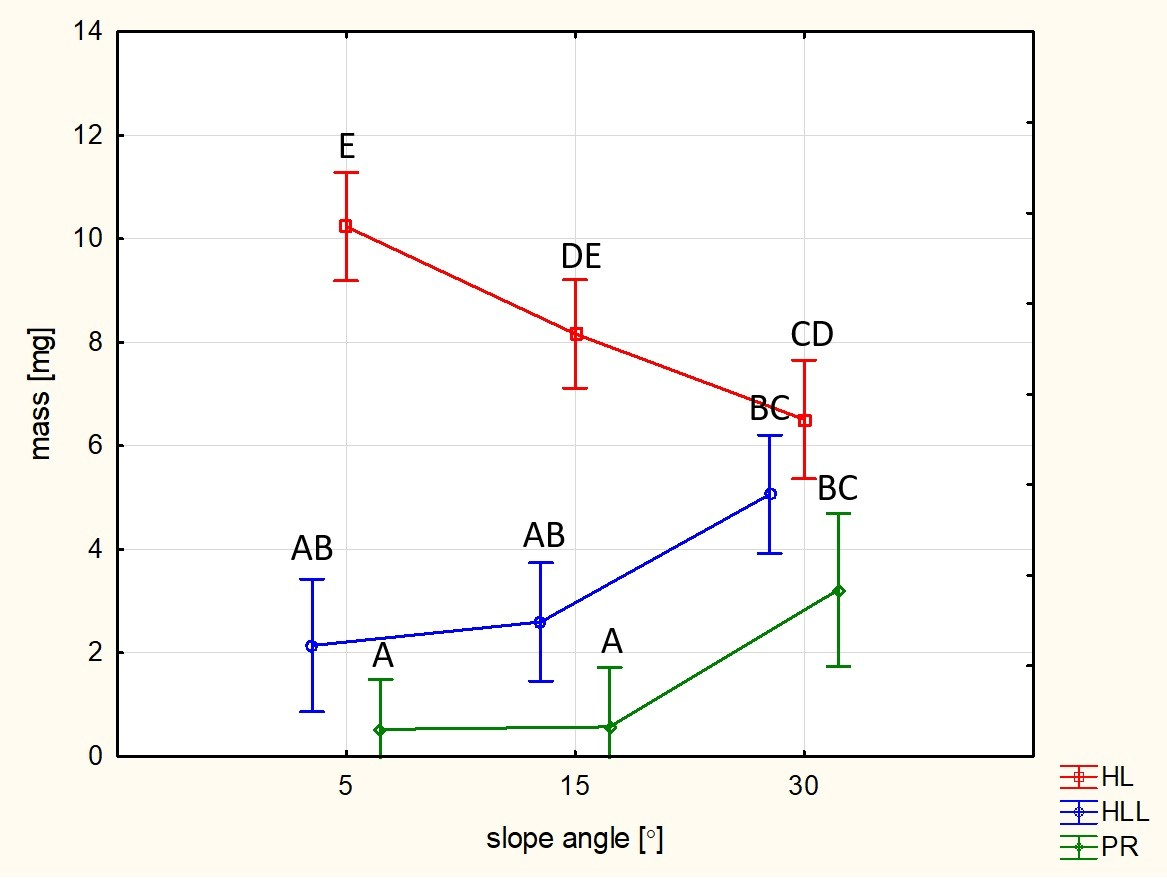

Supplement: S3 Fig — Symbols of soils: HL–Haplic Luvisol, HLL–Haplic Luvisol Loamic, PR–Protic Regosol. The bars represent standard error and letters refer to the statistical comparison (the same letters–no statistically significant differences). (TIF) [file pone.0262203.s003.tif]

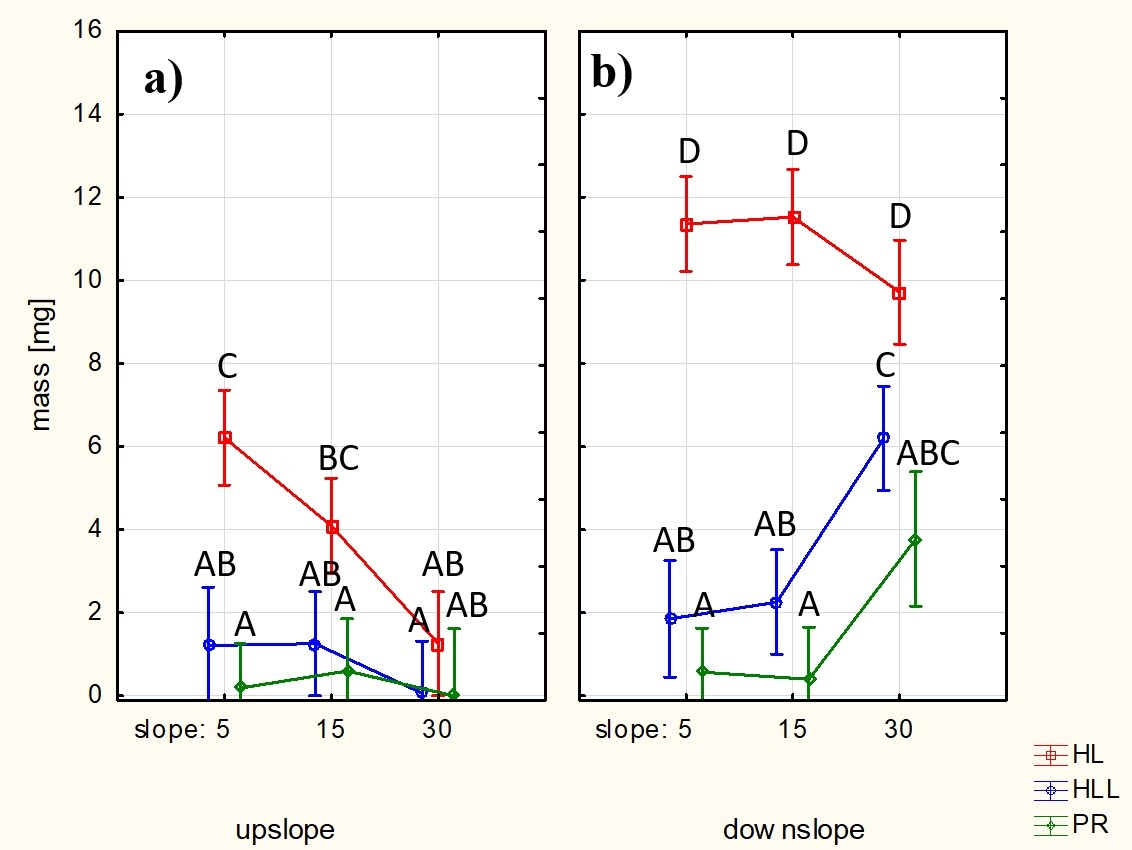

Supplement: S4 Fig — Mass of total ejected material in upslope (a) and downslope (b) directions on moistened soil samples with different slope angles. Symbols of soils: HL–Haplic Luvisol, HLL–Haplic Luvisol Loamic, PR–Protic Regosol. The bars represent standard error and letters refer to the statistical comparison (the same letters–no statistically significant differences). The statistical analysis allows the comparison of both graphs. (TIF) [file pone.0262203.s004.tif]

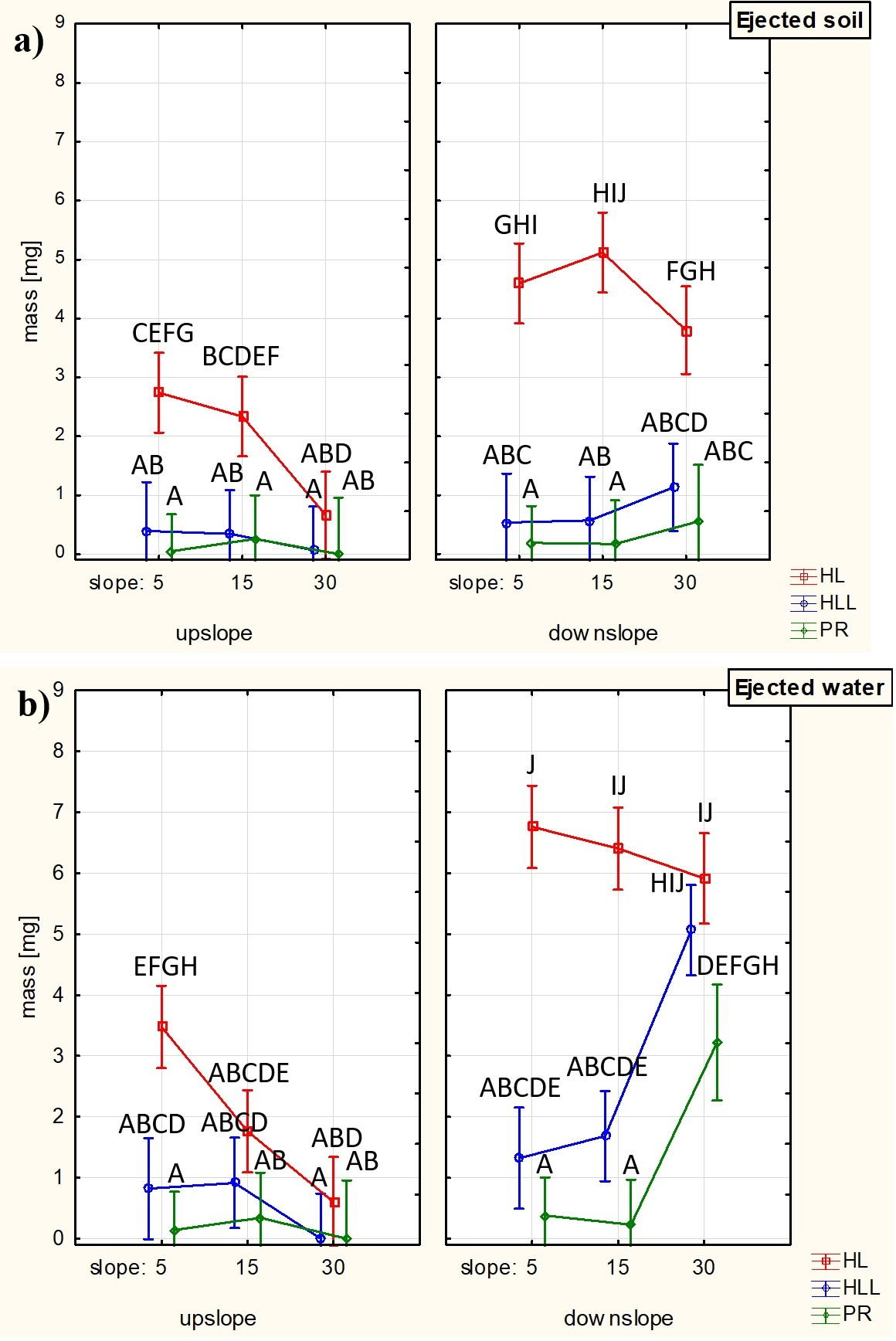

Supplement: S5 Fig — Mass of separate ejected soil (a) and ejected water (b) in upslope and downslope directions on moistened soil samples with different slope angles. Symbols of soils: HL–Haplic Luvisol, HLL–Haplic Luvisol Loamic, PR–Protic Regosol. The bars represent standard error and letters refer to the statistical comparison (the same letters–no statistically significant differences). The statistical analysis allows the comparison of all visible graphs. (TIF) [file pone.0262203.s005.tif]

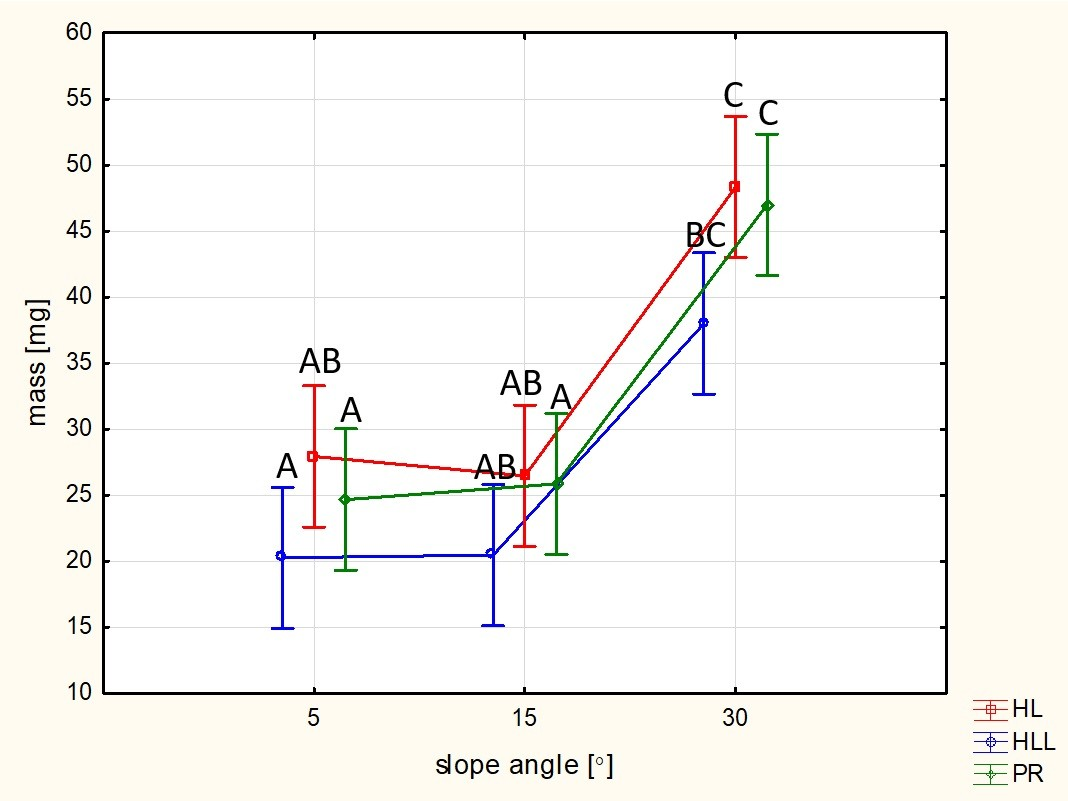

Supplement: S6 Fig — Symbols of soils HL–Haplic Luvisol, HLL–Haplic Luvisol Loamic, PR–Protic Regosol. The bars represent standard error and letters refer to the statistical comparison (the same letters–no statistically significant differences). (TIF) [file pone.0262203.s006.tif]

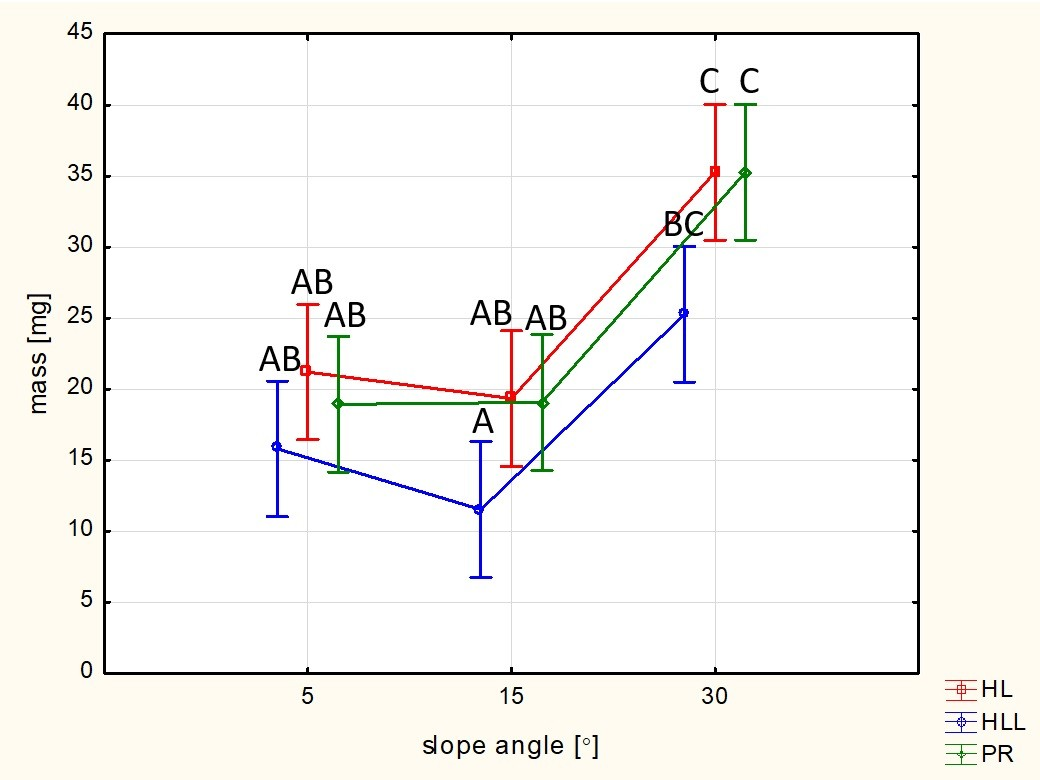

Supplement: S7 Fig — Symbols of soils: HL–Haplic Luvisol, HLL–Haplic Luvisol Loamic, PR–Protic Regosol. The bars represent standard error and letters refer to the statistical comparison (the same letters–no statistically significant differences). (TIF) [file pone.0262203.s007.tif]

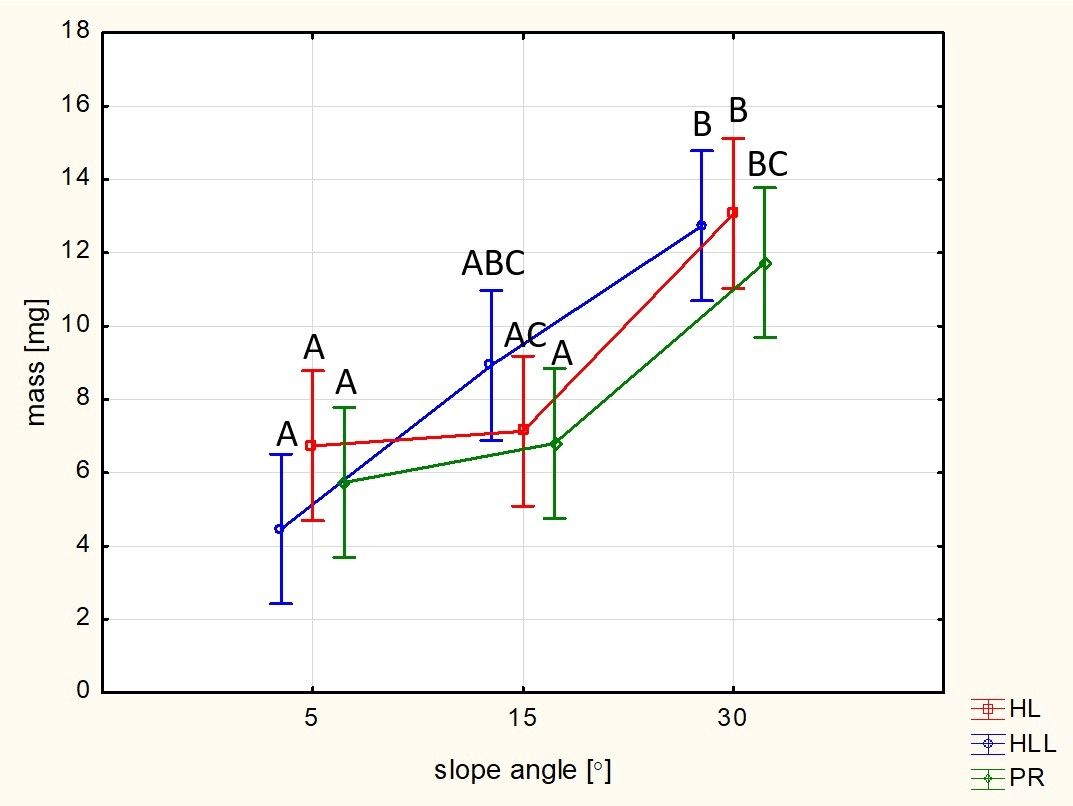

Supplement: S8 Fig — Symbols of soils: HL–Haplic Luvisol, HLL–Haplic Luvisol Loamic, PR–Protic Regosol. The bars represent standard error and letters refer to the statistical comparison (the same letters–no statistically significant differences). (TIF) [file pone.0262203.s008.tif]

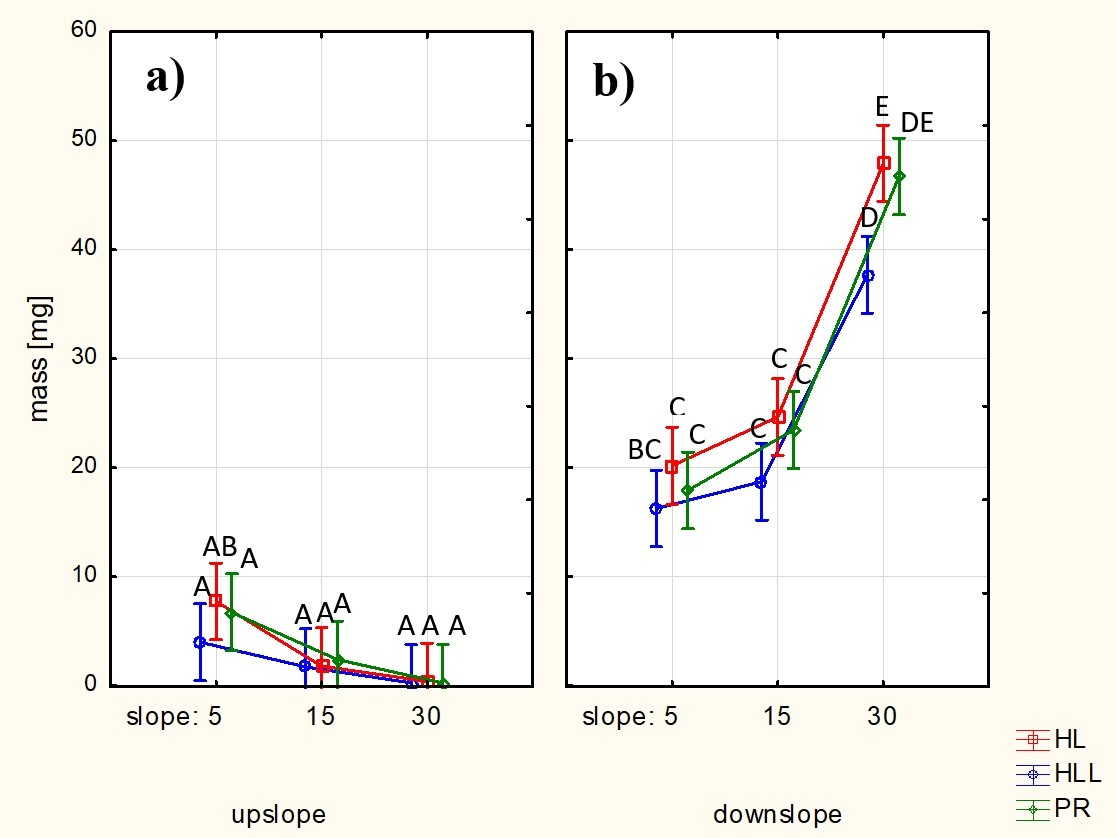

Supplement: S9 Fig — Mass of total ejected material in upslope (a) and downslope (b) directions on dry soil samples with different slope angles. Symbols of soils: HL–Haplic Luvisol, HLL–Haplic Luvisol Loamic, PR–Protic Regosol. The bars represent standard error and letters refer to the statistical comparison (the same letters–no statistically significant differences). The statistical analysis allows the comparison of both graphs. (TIF) [file pone.0262203.s009.tif]

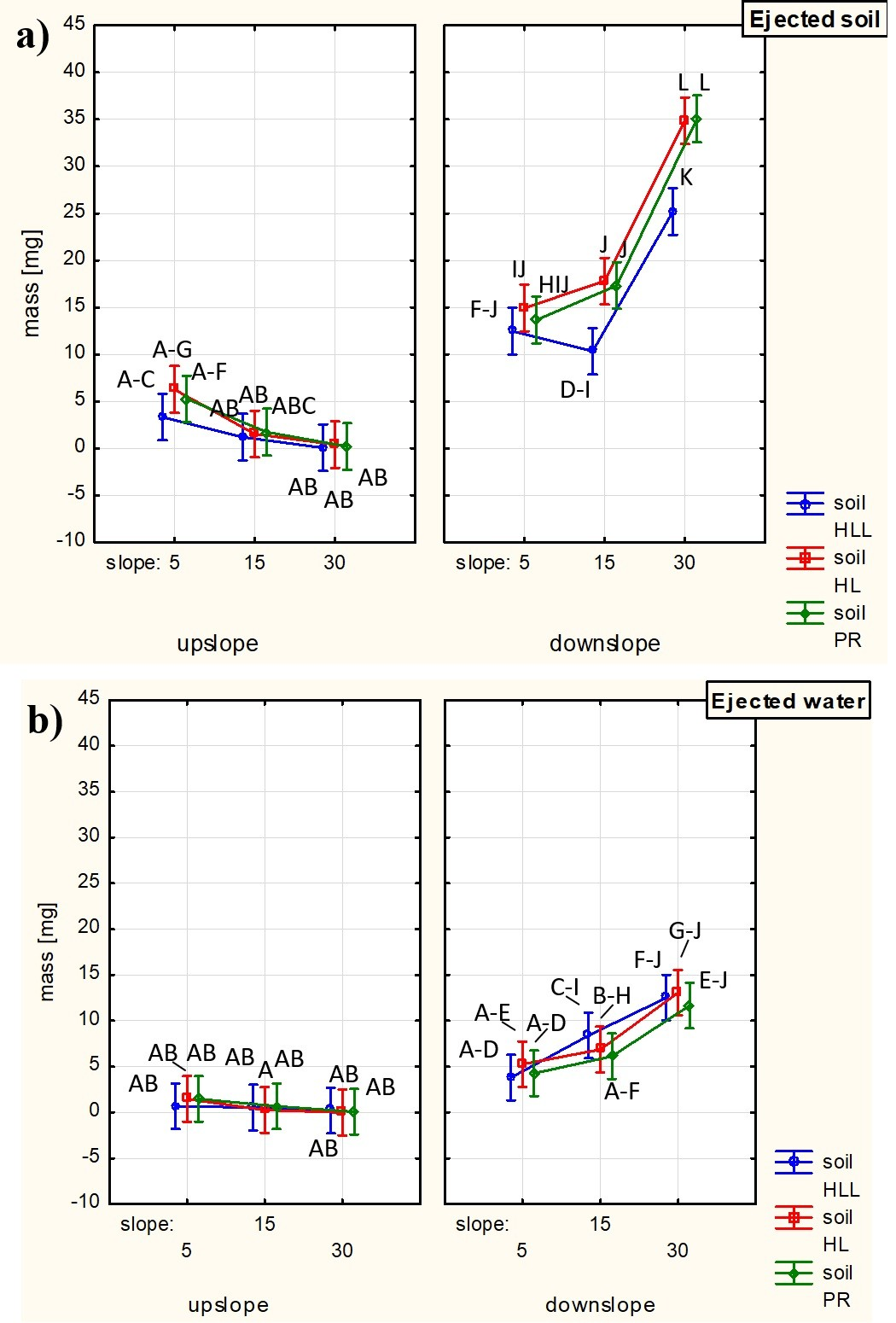

Supplement: S10 Fig — Mass of separate ejected soil (a) and ejected water (b) in the upslope and downslope directions on dry soil samples with different slope angles. Symbols of soils: HL–Haplic Luvisol, HLL–Haplic Luvisol Loamic, PR–Protic Regosol. The bars represent standard error and letters refer to the statistical comparison (the same letters–no statistically significant differences). The statistical analysis allows the comparison of all visible graphs. (TIF) [file pone.0262203.s010.tif]

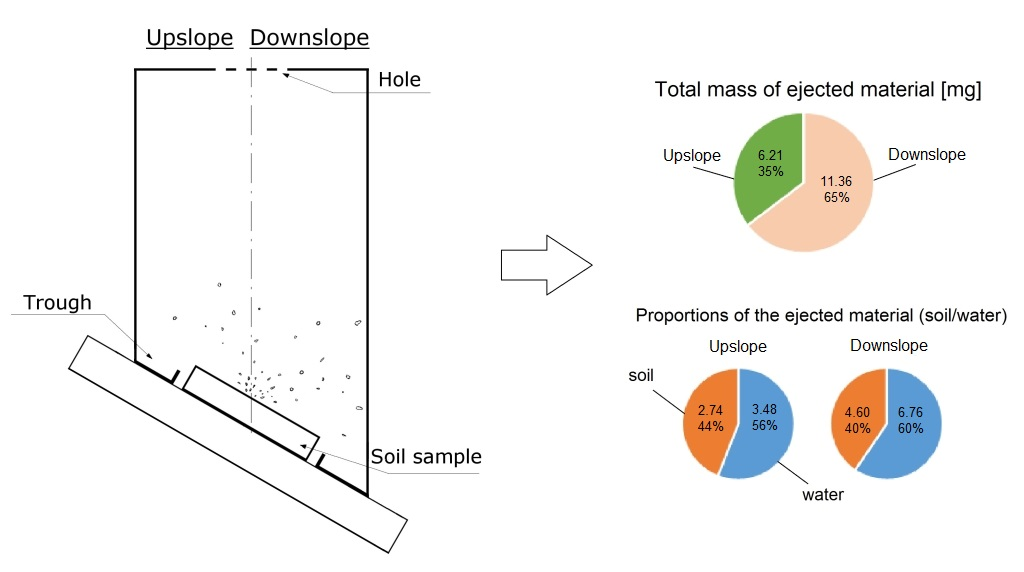

Supplement: S1 Graphical abstract — (TIF) [file pone.0262203.s011.tif]
